# Senior stakeholder views on policies to foster a culture of openness in the English National Health Service: a qualitative interview study

**DOI:** 10.1177/0141076818815509

**Published:** 2018-12-03

**Authors:** Graham Paul Martin, Sarah Chew, Mary Dixon-Woods

**Affiliations:** 1 The Healthcare Improvement Studies Institute, University of Cambridge, Cambridge, CB2 0AH, UK; 2 Center for Medicine, University of Leicester, Leicester, LE1 7RH, UK

**Keywords:** Employee voice, organisational silence, Freedom to Speak Up, organisational culture, National Health Service, England

## Abstract

**Objectives:**

To examine the experiences of clinical and managerial leaders in the English healthcare system charged with implementing policy goals of openness, particularly in relation to improving employee voice.

**Design:**

Semi-structured qualitative interviews.

**Setting:**

National Health Service, regulatory and third-sector organisations in England.

**Participants:**

Fifty-one interviewees, including senior leaders in healthcare organisations (38) and policymakers and representatives of other relevant regulatory, legal and third-sector organisations (13).

**Main outcome measures:**

Not applicable.

**Results:**

Participants recognised the limitations of treating the new policies as an exercise in procedural implementation alone and highlighted the need for additional ‘cultural engineering’ to engender change. However, formidable impediments included legacies of historical examples of detriment arising from speaking up, the anxiety arising from increased monitoring and the introduction of a legislative imperative and challenges in identifying areas characterised by a lack of openness and engaging with them to improve employee voice. Beyond healthcare organisations themselves, recent legal cases and examples of ‘blacklisting’ of whistle-blowers served to reinforce the view that giving voice to concerns was a risky endeavour.

**Conclusions:**

Implementation of procedural interventions to support openness is challenging but feasible; engineering cultural change is much more daunting, given deep-rooted and pervasive assumptions about what should be said and the consequences of mis-speaking, together with ongoing ambivalences in the organisational environment about the propriety of giving voice to concerns.

## Introduction

Although the insights from the ‘front line’ or ‘sharp end’ of organisations are an important resource for detecting problems, learning and improvement, organisations often struggle to encourage ‘employee voice’ and to respond appropriately.^1^ This is a particular challenge in healthcare, where the importance of speaking up about concerns has been repeatedly demonstrated. In the United Kingdom, for example, the first modern inquiry into National Health Service (NHS) failings arose from concerns reported by a nursing assistant at Ely hospital in Cardiff.^2^ The NHS is far from unique in experiencing these problems: similar difficulties in eliciting and making use of concerns of those at the sharp end of care have also been implicated in problems of quality and safety in health systems globally.^3^

Latterly, voice in the healthcare system has become a prominent focus of government policy.^4,5^ In England, this attention has been driven in particular by a high-profile failings in care at Mid Staffordshire NHS Foundation Trust. Led by Sir Robert Francis QC, a public inquiry into events at the hospital,^6^ and a more wide-ranging review of *Freedom to Speak Up*,^7^ suggested that voice in healthcare organisations was inhibited by individuals’ fear of repercussions and by perceptions of futility. In response, the government declared its intention to take steps to foster openness, defined in the Francis inquiry as ‘enabling concerns to be raised and disclosed freely without fear, and for questions to be answered’.^6^

Some of these steps have taken a procedural form. They include initiatives such as legal protection for ‘whistle-blowers’ and revisions to guidance on reporting and investigating serious patient safety incidents.^8,9^ Organisations have also been mandated to appoint ‘Freedom to Speak Up Guardians’ (conduits for concerns about facilities, quality of care, or colleagues’ behaviour) and must fund the role themselves. An additional, distinctive feature of policy on openness is its emphasis on the importance of attending to the *culture* of the NHS as a whole, and the extent to which it ‘actively promotes the benefits of openness and transparency’.^10^ ‘Chief Executives and Boards’, states one policy document, ‘should promote a culture of openness’, seeking to embed the policies by translating regulatory requirements into cultural change.^10^

The academic literature supports the notion that organisational-cultural influences have a critical impact on voice.^11–13^ Research demonstrates the importance of features of the organisational environment in encouraging or inhibiting voice, for example, the role of psychological safety in reducing fear of adverse consequences associated with decisions to speak up.^1,14,15^ Studies have also identified the relevance of heuristic schemas such as ‘implicit voice theories’ about when it is appropriate to speak, which may result in self-censorship and habituated silence.^11,12^ Barriers to voice, therefore, may have their roots more in an organisation's cultural cues, and in entrenched assumptions about appropriate behaviour, than in explicit policies or the dynamics of specific opportunities to speak. But how to realise a policy commitment to *cultural change* (rather than procedural implementation) of the kind necessary to address these barriers remains an important challenge and one that we address in this article? We report findings from a recent interview-based study that sought to examine the experiences of clinical and managerial leaders in the English healthcare system who were charged with implementing the policy goals of openness.

## Methods

We conducted semi-structured interviews with senior leaders (including clinicians and administrators) in English healthcare organisations, along with policymakers, representatives of regulatory bodies and individuals from relevant medico-legal and third-sector organisations, as part of a wider mixed-methods policy evaluation.

Senior leaders were identified through a mixture of random, purposive and snowball sampling techniques. With a view to securing representativeness, a randomly generated selection of acute trusts (20), community and mental healthcare trusts (10) and ambulance trusts (5) was contacted to identify potential participants. In parallel, we purposively sampled four organisations that had experienced problems with openness, as indicated by regulatory intervention and/or media coverage. Finally, we asked participants to suggest colleagues within or beyond their organisations who might be able to offer insights relating to our research questions. Wider stakeholders – such as policymakers and representatives of regulatory, third-sector and medico-legal organisations – were identified purposively in consultation with a stakeholder reference group, with snowball sampling again supplementing this initial list.

Data collection occurred between July 2017 and January 2018. Interviews were guided by a topic guide based on a literature review and discussion among the authors, collaborators, the stakeholder reference group and a patient and public involvement group. The guide was intended to elicit participants' in-depth understanding of relevant policies, including the clarity and unity of direction they provided, the process of implementing them and incentives and disincentives to increase openness. Interviews averaged 40 min and were audio-recorded and transcribed verbatim.

Supported by NVivo 11, we used an approach derived from the constant-comparative method for analysis.^16^ Interview transcripts were read independently by GPM and SC, who coded the data for high-level themes derived from the evaluation brief and academic literature, and themes identified inductively from close reading of the data. We modified, developed and amalgamated codes as we read and re-read data sources. Coding was accompanied by ongoing discussion among the authors. GPM then drafted an integrated analysis of the findings in relation to the research questions above, which was developed and agreed by all authors, and is presented below.

## Findings

We interviewed 18 participants from acute hospitals (denoted Ac in data excerpt attributions), 17 from community and mental healthcare trusts (MH), and three from ambulance trusts (Am). Participants came from 16 acute trusts and one non-NHS provider of acute services, 11 community and mental healthcare trusts and three ambulance trusts: in total, 31 different organisations were represented. We also interviewed 13 wider stakeholders, bringing the total number of interviewees to 51. NHS-employed participants largely occupied senior management positions but also included eight Freedom to Speak Up Guardians of varying seniority.

### The challenge of encouraging voice

The need for greater openness was broadly acknowledged and accepted by participants. Many cited the Francis inquiry as a driver for change, but some noted that change in their organisations had started earlier, often in response to a serious local incident. Participants recognised that organisations were likely to feature ‘dark spots’^17^ of poor performance and practice, where lack of organisational knowledge or reluctance to speak up obscured poor care.

My perception is that we're probably doing a lot better than other places, but you don't know what you don't know. If staff are reluctant to come to anybody and raise their concerns, how do you know that? How do you benchmark it? (Ac06)

Although the aspiration for openness was welcomed, the challenges associated with encouraging voice were seen as daunting. Participants described fear of speaking up as having complex origins, relating to both perception and reality.

First, implicit voice theories^12^ were seen as influential. Participants described how their colleagues associated speaking up sometimes with extreme consequences, such as job loss or litigation, and sometimes with less dramatic but nonetheless important fears, such as difficult interactions with managers or being seen as the cause of trouble or extra work. A closed culture was seen as the natural consequence of such assumptions.

If people put their head above the parapet, [they fear] that they will suffer themselves, either through being isolated or victimized, or – worst-case scenario – that they would suffer by losing their job, because that has happened in places up and down the country. And the reputation of whistleblowing is very much [that] you're taking a risk by doing this. (MH12)

A second reported influence on a closed culture was the behaviours of leaders who either failed to listen or actively suppressed voice, inducing silence through aggression or indifference.

Sometimes you get very longstanding management teams within a specific directorate, [and] they can stop hearing. […] And people as a consequence feeling they can't speak up: it's not the norm. Everything's alright because nobody's said anything. (MH10)

One of my team raised a concern to the previous chief executive, and was told not to bring a problem to the table. He didn't want to hear. […] It has an impact over time. (Ac11)

Finally, participants saw closed cultures as arising when staff simply did not notice what was going wrong. In some settings, the prevailing cultural disposition – that is, the taken-for-granted beliefs and behaviours of a unit^18^ – was not to question. Staff did not speak up because they did not perceive the need to do so.

[We had issues with] a small community hospital, completely off our radar, low level of complaints, care of older people, people not speaking up in that particular environment. So quite shocking to discover. (Ac04)

Such deficits of openness were seen as most likely to occur in isolated groups, less exposed to broader norms and inclined to be more inward-looking. Participants reported that these groups might also be among those most difficult to support in change towards openness.

### Promoting openness

Participants described taking seriously the goal of promoting openness, often giving accounts of concerted organisational efforts to implement regulatory requirements and of cultural work to reshape organisational norms through strengthening of relationships and creating a narrative of collective accountability.

One set of tasks associated with realising openness was largely procedural in nature. Participants described, for example, their work to appoint Freedom to Speak Up Guardians and prepare clear organisational statements that explicitly encouraged voice. Achieving these procedural tasks was not straightforward but could at least be structured and managed through clear operational plans.

We took the recommendations, we went through and picked out all of those that could possibly apply to a non-acute provider. And we grouped them in areas that fitted into work that we were doing, and monitored against them. (MH02)

Much more challenging were the cultural tasks of reshaping organisational norms, values and behaviours towards openness. Many of the actions described by participants appeared to target directly the challenges that they saw as contributing to closed cultures.

First, they sought to create relationships between management and staff characterised by trust and confidence. They emphasised the need for clarity and consistency about the mutual obligations and expectations of the employee–management relationship and to reassure staff that punitive intent would play no role in responses to concerns being raised. Accordingly, they stressed the need to ensure alignment between espoused and enacted values by senior leaders.

There is a tentative period where people are watching whether you are going to do what you said, and I think having a set of values, that the leadership doesn't lead, is a kind of anti-value, really: it is worse than useless. (Ac03)

In particular, they sought to convince colleagues that giving voice to concerns was a worthwhile activity that would deliver benefits at the sharp end, not just in administrators' reports to their superiors. This meant efforts to establish openness as the default orientation and to position conversations about shortcomings in care as a route to collaborative improvement rather than hierarchical accountability.

The most important bits around openness and transparency, where the greatest success has been, [is] just by the executive team – so me and my colleagues – out in the patch, walking, understanding the issues, meeting the staff, so we can see the problems that they're facing. I think that's the biggest change. (MH08)

Second, participants sought to create a narrative of collective accountability that both fostered a sense of being ‘on the same side’ and emphasised shared values. Strategies for creating and sustaining dialogue included use of Schwartz rounds (a method by which staff from all levels can reflect on the emotional aspects of their roles, with a view to legitimising and normalising openness). Participants also described their work to take collective ownership of problems of quality and safety, modelling openness and embracing vulnerability rather than loading responsibility onto the sharp end:

[Chief executive] has a blog in our intranet, and it is completely uncensored; we don't have any time lag between comments to be able to censor. Some people sail very close to the wind but nonetheless we support free expression. […] If we hear things that make us feel uncomfortable, all good. (MH03)

Third, participants described the importance of meaningful responses to concerns. In this model, problem-solving was a key responsibility of leaders, including closing the feedback loop by ensuring that those who raised issues were informed of progress.

We’ve done other things; where they said, ‘This is not right’, we’ve bought a piece of kit. So whenever we go around, we have a little pot of money that we can actually go into and say, ‘Well this is to help get it sorted’. So I think people recognise that we want to go out there and hear. (Ac01)

All in all, these efforts focused on seeking to establish willingness to speak up as the default orientation, by sharing the burdens and benefits of greater openness between the blunt and sharp ends.

### Frustrating a culture of openness?

Participants were cautious about the prospects for their efforts in improving openness. Both direct experiences and shared lore about the risks of speaking up carried enduring weight and informed implicit voice theories. Procedural interventions, they felt, might be futile in the face of deeply rooted assumptions about organisational behaviour but even efforts to intervene in organisational culture could flounder.

There are certain areas where people feel more vulnerable than in others. And depending on previous experiences, even quite historical, if there's been a particularly significant event and there's been any kind of staff disciplinaries on the back of that, that legacy might still be there in a team, sometimes many years later. (MH14)

This meant that no strategy was uniformly effective. Participants found that their organisations were not homogenous with respect to openness but varied area-by-area, team-by-team. Organisations that operated across multiple sites were seen to be at particular disadvantage in trying to inculcate a common culture that normalised openness. Just as some parts of their organisations had cultures that appeared more closed, organisational units varied in their response to efforts to breed openness.

We're very geographically challenged, because [Town A] and [Town B] are 45 miles apart. […] To promote speaking up in everyday practice, business as usual, that's going to take a long time to embed. (Ac09)

Beyond localised ‘resistance’ to efforts to foster openness, a system-wide sense of vulnerability also remained, because many of the assumptions about the risks of speaking up still rang true. Whatever formal policy proclaimed, and however sincere individual organisations were in implementing it locally, the wider system still contained conflicting signals about the risks and benefits of openness. Widely publicised criminal convictions,^19^ along with cases of ‘blacklisting’ of whistle-blowers, sustained the message that openness was not risk free.

What happened recently with the paediatrician, and a couple of other cases of corporate manslaughter, or individuals: […] those kind of cases really risk people being open and honest. (Wider stakeholder 10)

If you talk to staff – and I know because staff tell me – there's still a bit of fear about being open. So however much they're reassured, there's still this belief that it may lead to being disciplined, or sanctions, or opportunities being limited. (Am01)

Furthermore, participants reported that some aspects of government policy perversely risked inhibiting openness. Alongside policies to promote voice, for example, the government had introduced a statutory ‘duty of candour’, obliging organisations and clinicians to acknowledge, apologise in writing for and learn from incidences of moderate or severe harm caused to patients.^9^ For those who failed to uphold this duty, punitive consequences could follow, including criminal prosecution.^20^ The legalistic language surrounding the duty could, some participants argued, lend further credence to the notion that openness was being forced upon the healthcare service, with a greater focus on blame than learning:

To make it a criminal offence, I think, was entirely wrong. […] Frightening people, by saying this is a statutory duty of candour, doesn't necessarily lead people to being more open. (MH02)

There was also a sense that some of the accountability requirements associated with openness risked subverting the substantive intent of the policies.

Each initiative has to be counted and double-counted, and monitored, and it is a distraction from what it's actually about. […] The requirement for more-or-less standard records to be kept about not only the nature of the concern, but the characteristics of the person raising the concern. […] It's not always appropriate to say, ‘Are you happy with the approach that I’ve taken?’ at that particular point. (MH05)

Overall, participants reported that the weight of the past, alongside ongoing developments in the present, could render their efforts at securing change fragile.

## Discussion

Our findings suggest some enthusiasm for openness initiatives among senior stakeholders across the English NHS. However, many recognised the limitations of treating the new policies as an exercise in procedural implementation alone. They understood that the initiatives were hard to sell to colleagues functioning in suboptimal conditions, or who had witnessed or heard about maltreatment of colleagues who had spoken up about concerns in the past. When the prevailing understanding was that speaking up remained a ‘high risk: low benefit act’,^21^ new procedures and the appointment of figures such as Freedom to Speak Up Guardians were seen as unlikely to provide reassurance.

Accordingly, senior stakeholders sought to supplement implementation of policies and roles with a sort of ‘cultural engineering’ to address reticence and assure their colleagues that giving voice to concerns would now be welcomed, not punished. They attempted to do this in ways that mapped well onto existing knowledge about leadership behaviours that empower and engage colleagues in improvement, such as senior management visibility, connecting proposed change to wider values and vision and finding common purpose.^22,23^ They sought to ensure that some of the advantages of an open culture accrued at the sharp end and to make openness useful in addressing everyday imperfections as well as ‘big-ticket’ problems. Achieving this involved ongoing dialogue between the blunt and sharp ends, focused on understanding over accountability, with a view to instilling openness as the default disposition.^17,24^

Participants argued that cultural work to engage, and to share the benefits and burdens of openness, was essential in underwriting any behavioural change in relation to voice. But they also acknowledged that this approach faced its own barriers. One was the heterogeneous character of their organisations: the parts that might most benefit from efforts to improve openness were often those that were difficult to identify as problematic, difficult to reach and difficult to influence. A second was that some characteristics of the policies risked reinforcing the view that voicing concerns was a risky activity. The monitoring activity that surrounded the Freedom to Speak Up Guardians, for example, meant that preoccupation with the letter of the law might undermine its spirit, converting it into a bureaucratic display of compliance or, worse still, a punitive threat. While policy documents stressed the importance of ‘learning not blaming’,^8^ the risk was that the very regulatory practices intended to support it might have the opposite effect.

Thus, senior stakeholders understood that they needed to go beyond legalistic reassurances of protection for those who spoke up, and even beyond efforts to foster psychological safety that might embolden staff to give voice to concerns,^11^ towards generating environments where the value of openness was apparent. Given the mixed signals of the wider system, however, and the diversity of experiences and expectations within organisations, our analysis suggests that participants may need to attend to creating an organisational infrastructure that might reinforce the well-meaning words intended to show that the blunt and sharp ends could both benefit from greater openness. There was little sign of the significant, programmatic investments in structured processes for support, development and intervention that research suggests have underpinned changes in organisational culture elsewhere,^25–27^ especially for addressing the challenges of diverse micro-cultures within healthcare organisations.^18^ While efforts to lead by example and ensure that benefits accrue at the sharp end are surely necessary, it is perhaps doubtful whether they are sufficient to secure sustained cultural change around openness when messages about its risks and rewards remain mixed.

For other systems looking to emulate the policies and initiatives developed in the English NHS to foster openness, our findings suggest two lessons. First, work is required to make aspirations of openness relevant to sharp-end clinicians working in pressured environments, for whom compromises and workarounds are a taken-for-granted feature of routine work, and who may see such interventions primarily as blame-allocation devices.^28^ This means that actions must match words, especially in environments where initiatives purportedly intended to prompt learning and improvement have a tendency to metamorphose into tools of performance management.

Second, and more broadly, the word ‘openness’ is perhaps too passive a term to describe what is desired here. A major barrier to openness identified by participants was not concealment or opacity among their colleagues but rather a kind of normalised incuriosity. Intervening in such contexts is challenging: established routines of explanation and rationalisation may over time become institutionalised as legitimate ways of dealing with problems; it is difficult to disrupt these routines without the disruption itself being deemed deviant.^29,30^ Open cultures therefore require active nurturing by those seeking to foster them, to imbue a state of continued, reflexive inquiry and self-questioning. This is not something that can be achieved by policy implementation alone. However, it may also require more than fine words and symbolic deeds on the part of leaders, especially given the shadow of history and the equivocal signals of the present.

Our study has limitations. Participants self-selected in their response to requests for interview. It is plausible that they represented organisations that were more forward-thinking in their approach to fostering openness; indeed, wider stakeholder participants affirmed that some organisations were less advanced in implementing openness policies. Despite our partly random sampling strategy, therefore, it should not be assumed that our findings are nationally representative. Furthermore, we have only interview accounts of participants' views and of their organisations and colleagues' behaviour, and we have no measure of the impact of the approaches they saw as more or less successful.

## Conclusion

Calls to improve employee voice pose challenges for senior stakeholders. While implementation of procedure is possible, engineering cultural change is daunting, given deep-rooted and pervasive assumptions about what should be said and the consequences of misspeaking, together with ongoing ambivalences in the organisational environment about the propriety of giving voice to concerns. Visible efforts to reframe the relationship between blunt and sharp ends of organisations seem a promising approach, but it is not clear that such endeavours will succeed in the absence of an infrastructure that underwrites positive words with consistent organisational action.

## Declarations

### Guarantor

GPM.

### Provenance

Not commissioned; peer-reviewed by Jackie Bridges and Sondus Hassounah.
